# Automated detection of moderate and large pneumothorax on frontal chest X-rays using deep convolutional neural networks: A retrospective study

**DOI:** 10.1371/journal.pmed.1002697

**Published:** 2018-11-20

**Authors:** Andrew G. Taylor, Clinton Mielke, John Mongan

**Affiliations:** 1 Department of Radiology and Biomedical Imaging, University of California, San Francisco, San Francisco, California, United States of America; 2 Center for Digital Health Innovation, University of California, San Francisco, San Francisco, California, United States of America; Johns Hopkins University, UNITED STATES

## Abstract

**Background:**

Pneumothorax can precipitate a life-threatening emergency due to lung collapse and respiratory or circulatory distress. Pneumothorax is typically detected on chest X-ray; however, treatment is reliant on timely review of radiographs. Since current imaging volumes may result in long worklists of radiographs awaiting review, an automated method of prioritizing X-rays with pneumothorax may reduce time to treatment. Our objective was to create a large human-annotated dataset of chest X-rays containing pneumothorax and to train deep convolutional networks to screen for potentially emergent moderate or large pneumothorax at the time of image acquisition.

**Methods and findings:**

In all, 13,292 frontal chest X-rays (3,107 with pneumothorax) were visually annotated by radiologists. This dataset was used to train and evaluate multiple network architectures. Images showing large- or moderate-sized pneumothorax were considered positive, and those with trace or no pneumothorax were considered negative. Images showing small pneumothorax were excluded from training. Using an internal validation set (*n* = 1,993), we selected the 2 top-performing models; these models were then evaluated on a held-out internal test set based on area under the receiver operating characteristic curve (AUC), sensitivity, specificity, and positive predictive value (PPV). The final internal test was performed initially on a subset with small pneumothorax excluded (as in training; *n* = 1,701), then on the full test set (*n* = 1,990), with small pneumothorax included as positive. External evaluation was performed using the National Institutes of Health (NIH) ChestX-ray14 set, a public dataset labeled for chest pathology based on text reports. All images labeled with pneumothorax were considered positive, because the NIH set does not classify pneumothorax by size. In internal testing, our “high sensitivity model” produced a sensitivity of 0.84 (95% CI 0.78–0.90), specificity of 0.90 (95% CI 0.89–0.92), and AUC of 0.94 for the test subset with small pneumothorax excluded. Our “high specificity model” showed sensitivity of 0.80 (95% CI 0.72–0.86), specificity of 0.97 (95% CI 0.96–0.98), and AUC of 0.96 for this set. PPVs were 0.45 (95% CI 0.39–0.51) and 0.71 (95% CI 0.63–0.77), respectively. Internal testing on the full set showed expected decreased performance (sensitivity 0.55, specificity 0.90, and AUC 0.82 for high sensitivity model and sensitivity 0.45, specificity 0.97, and AUC 0.86 for high specificity model). External testing using the NIH dataset showed some further performance decline (sensitivity 0.28–0.49, specificity 0.85–0.97, and AUC 0.75 for both). Due to labeling differences between internal and external datasets, these findings represent a preliminary step towards external validation.

**Conclusions:**

We trained automated classifiers to detect moderate and large pneumothorax in frontal chest X-rays at high levels of performance on held-out test data. These models may provide a high specificity screening solution to detect moderate or large pneumothorax on images collected when human review might be delayed, such as overnight. They are not intended for unsupervised diagnosis of all pneumothoraces, as many small pneumothoraces (and some larger ones) are not detected by the algorithm. Implementation studies are warranted to develop appropriate, effective clinician alerts for the potentially critical finding of pneumothorax, and to assess their impact on reducing time to treatment.

## Author summary

**Why was this study done?:**

- Pneumothorax (collapse of the lung due to air in the chest) can be a life-threatening emergency.
- Delays in identifying and treating serious pneumothorax can result in severe harm to patients, including death.
- Pneumothorax is often detected by chest X-ray, but delays in review of these images (particularly at hours of lower staffing, such as overnight) can lead to delay in diagnosis and treatment.
- Prioritization of images that are suspected to show a pneumothorax for rapid review may result in earlier treatment of pneumothorax.

**What did the researchers do and find?:**

- We developed computer algorithms that scan chest X-rays and flag images that are suspicious for containing a moderate or large pneumothorax.
- These algorithms “learned” to identify moderate- and large-sized pneumothorax by training on a large set of both positive and negative chest X-rays.
- We created the training set of images by asking board-certified radiologists to label each image for the presence or absence of pneumothorax, as well as their estimate of pneumothorax size.
- After training, we tested the performance of the algorithms on a similar collection of labeled X-rays that had never been seen by the algorithms and analyzed their success at detecting images showing pneumothorax, without any human guidance.
- We found that our algorithms were able to detect the majority (80%–84%) of images showing a moderate or large pneumothorax, while correctly categorizing 90% or more of images without pneumothorax as “negative.” When we included small pneumothoraces in our test set, performance declined, as expected because the algorithms had not been trained on images with small pneumothoraces.
- When testing our algorithms using images acquired outside our hospital, performance declined compared with our internal testing. However, the tests of the external dataset were not exactly comparable to our internal tests: small pneumothoraces could not be excluded from the evaluation because labels in the external dataset did not include size, and labels were assigned by computer interpretation of clinical reports rather than radiologists reevaluating the images, limiting the accuracy of the labels.

**What do these findings mean?:**

- Computer algorithms, given enough high-quality training data, are capable of detecting pneumothorax on a chest X-ray with sufficient accuracy to help prioritize images for rapid review by physicians.
- Algorithms like these could potentially be used by radiologists as a tool to increase the speed with which a serious pneumothorax is detected, even at times of lower staffing, when turnaround times are typically longer.
- Rapid detection and communication with treating physicians may result in faster treatment of pneumothorax, potentially reducing the harm of a serious medical problem.
- The transferability of our models to clinical settings outside the institution where the training images were acquired needs further validation. Although we evaluated the models against an external dataset, differences in the composition, curation, and labeling between the external data and our own make it difficult to interpret these external dataset results.

## Introduction

Pneumothorax can constitute a medical emergency since the presence of air within the pleural space outside the lung produces collapse of the lung and subsequent respiratory distress, especially in critically ill patients [1]. While the incidence of spontaneous pneumothorax in the United States is relatively low [2], pneumothorax is often associated with trauma [3], mechanical ventilation [4], and iatrogenic injury from procedures such as thoracentesis [5]. The use of adjunctive imaging has reduced this risk somewhat, but even with ultrasound guidance a recent meta-analysis estimated the rate of pneumothorax after thoracentesis to be approximately 4% [6]. Pneumothorax of a clinically significant size is often diagnosed with standard frontal plain film radiography; however, the accuracy of diagnosis is dependent on a number of factors including pneumothorax size, patient positioning, image quality, and variation in radiologist threshold for diagnosis, resulting in a mean sensitivity in the range of 83%–86% in studies assessing this [7–9]. Further, treatment is reliant on timely review of acquired images, both by the radiologist and the referring physician. A study of patients with pneumothorax in the intensive care unit (ICU) found that length of stay in intensive care was longer and the risk of progression to tension pneumothorax (a large pneumothorax that causes obstruction or restriction of blood flow to the heart, producing circulatory collapse) was higher for patients whose pneumothoraces were initially misdiagnosed; further, a significant risk factor for delay in diagnosis and misdiagnosis was development of pneumothorax outside of peak physician staffing hours [10].

Since current hospital practices may result in long worklists of radiology images to be read, particularly those acquired overnight or without accompanying clinical suspicion of a significant problem, an automated method of screening chest X-rays and prioritizing studies with positive findings for rapid review may reduce the delay in diagnosing and treating pneumothorax.

Deep convolutional neural networks—a class of machine learning models that has found widespread application in computer vision and image classification tasks [11]—are increasingly being utilized in radiology and medical image analysis [12,13]. While these models can produce highly accurate results, they require large and well-curated training datasets in order to achieve acceptable performance on tasks where there is significant visual heterogeneity, as one might expect in a sample of chest X-rays obtained in clinical settings varying from outpatient clinics to inpatient ICUs, and with patients suffering from myriad illnesses.

Our objective was to create a large, human-annotated dataset of chest X-rays, relatively enriched in moderate and large pneumothoraces, and to use this set to train a deep convolutional neural network to identify these larger, potentially emergent pneumothoraces with performance suitable for prioritizing studies for rapid review under circumstances where turnaround times might be longer than usual, such as overnight or on weekends. We felt that a system for prioritization would require different target performance characteristics than would a system designed to be relied upon as a sole mechanism for diagnosis. In the latter, sensitivity would likely be the highest priority in order to avoid missing a potentially important finding, and specificity would likely decline as a result, increasing the false-positive rate. We felt that since a prioritization algorithm benefits from the “backup” of human radiologist review for all images, emphasis might be better placed on specificity so as to keep the false-positive rate low. This would reduce alert fatigue for the radiologist who is receiving these images flagged for priority review, and who might ignore the results of the algorithm if the detection of 1 true-positive study required review of dozens of false-positive cases.

## Methods

This study, compliant with the Health Insurance Portability and Accountability Act of 1996, was approved by the institutional review board of our institution. The study was granted a consent waiver due to its retrospective design and minimal risk categorization.

### Image extraction, anonymization, and annotation

Candidate images for analysis were identified by searching our clinical report database for chest X-rays with a clinical report finding of pneumothorax using mPower (Nuance Communications, Burlington, MA). Candidate chest X-rays of adult patients were obtained from 1 January 2006 to 31 December 2016. Candidate images for inclusion in the positive group were identified by using the search terms “small pneumothorax,” “trace pneumothorax,” “moderate pneumothorax,” and “large pneumothorax” with mPower’s standard filters in place to minimize negative occurrences. Candidate images for inclusion in the negative group were identified both by searching for negative phrases such as “no pneumothorax” and by including X-rays from the same time period that did not meet the criteria for inclusion in the positive group. This dual approach was used to ensure that the negative class did not only contain images where the report specifically stated “no pneumothorax.” No exclusion criteria were applied to the candidate negative images other than absence of pneumothorax; this ensured that these images would include the range of techniques, patient classes, and non-pneumothorax imaging classes expected to be encountered in clinical practice. The candidate images were then retrieved in preparation for visual reannotation of pneumothorax presence and size. Candidate studies were bulk extracted in DICOM format from the clinical PACS (picture archiving and communication system) using a custom-built automated image retrieval system, and metadata were stored in a SQL database.

### Resolution downsampling

The full resolution DICOM files were initially converted to 8-bit JPEG grayscale images using the dcmj2pnm utility of DCMTK (https://www.dcmtk.org, version 3.6.1). LUT transformation was performed using the software’s default min-max algorithm, discarding extreme values. These JPEG images were then downsampled with the ImageMagick convert utility (http://www.imagemagick.org, version 6.8.9.9-7ubuntu5.9) for use in the neural network. Images were downsampled to 512 × 512, because we found it was difficult for the annotating radiologists to see the pneumothoraces at the 256 × 256 or 224 × 224 resolutions commonly used with image-recognition convolutional neural networks. All images were downsized using the default (Lanczos) resize filter and were squashed to achieve a square aspect ratio.

### Annotation

All studies in the dataset were annotated after visual inspection by 2 board-certified and 4 board-eligible radiologists, all with a minimum of 4 years’ experience. Annotation was performed using custom-built visualization and annotation software, with each study receiving 2 annotations (right and left lung) across the following categories of pneumothorax size: none, trace, small, moderate, and large. Annotators were given a rubric describing the definitions for each of these classifications (see S1 Text and S1 Fig for additional information). Resulting annotations were stored in the SQL database. All images that were initially annotated as positive for pneumothorax, and all images in the separate held-out test set were then reannotated by multiple annotators in a blinded fashion, and majority consensus was then used to arrive at a single pneumothorax size classification for each lung. For each image, a single global annotation was then determined by selecting the largest pneumothorax size annotated for either lung. All training and model performance evaluation was based on a single pneumothorax annotation per image.

### Frontal image selection

Many studies within our database contained multiple images (e.g., posteroanterior and lateral). In many cases, information indicating the body part or view was not present in the DICOM header. Therefore, we used our previously developed chest radiograph orientation model [14] to classify images as either frontal, lateral, or other. For each annotated study used, we selected the image with the highest frontal chest score, above a minimum threshold of 0.8 probability.

### Dataset assembly

The dataset comprised 13,292 annotated studies, which were then shuffled and separated into a 70%/15%/15% training/validation/test set split. In this nomenclature, the validation set was used to evaluate model performance at the end of each epoch during training and for hyperparameter optimization, and the test set was a held-out set of images used for evaluation of the trained models, never seen by the algorithm during training or validation. Because images were treated individually, the datasets were kept disjoint by study, but not by patient. These sets were kept static so that all training experiments used the same sets of images for training and evaluation.

After the split, the training set had 2,214 positive and 7,095 negative images, the validation set had 456 positive and 1,537 negative images, and the test set had 437 positive and 1,553 negative images. Due to the low prevalence of pneumothorax expected in a general sample of chest X-rays, it was necessary to balance the training dataset to provide enough cases of pneumothorax to train the model. Therefore, each training minibatch was created using equal numbers of positive and negative images. In order to prioritize the detection of the most potentially clinically significant pneumothoraces, we defined positive images as only those labeled moderate or large pneumothorax in accordance with the research plan established during a design session prior to beginning this work (S2 Text). Images labeled as small pneumothorax were excluded from training. Trace pneumothoraces were considered to be negative cases for both training and the test set, since our definition of trace was a pneumothorax too small to be clearly seen. The primary intent of including trace pneumothoraces as negative studies in training was to increase the number of negative studies in which a chest tube is present. The goal of this was to avoid training the classifier to make positive predictions based on detecting a chest tube as a proxy for pneumothorax.

### Model implementation and models tested

We used the Keras (version 2.0.3, https://keras.io/) deep learning library on top of TensorFlow (version 1.2.1, Google) to implement convolutional neural network models. Training and test were performed on an NVIDIA DGX-1 using up to 8 Tesla P100 GPUs. We based our models on several standard network architectures implemented within Keras, including VGG16/19, Xception, Inception, and ResNet [15–18]. The convolutional layers of the architectures were completely unchanged, and we experimented with average pooling, max pooling, or flattening to pool the outputs of the final feature maps for each architecture. These were connected to new fully connected layers (fc1 and fc2), followed by a final output sigmoid that predicts pneumothorax class. We used a binary cross-entropy loss function. The sizes of the fully connected layers were optimized using random search. Models were tested using initialization from random weights as well as using transfer learning with models pretrained on the ImageNet Large Scale Visual Recognition Challenge datasets.

We used the drop-in streaming image augmentation pipeline within Keras to build and train all models. This system expands on the base dataset by generating images on the fly during training using a variety of elementary transforms. We allowed horizontal (but not vertical) image flipping, image zooming, shearing, and rotation. The maximum parameter ranges for all augmentation operations were explored through a hyperparameter search described below.

### Hyperparameter optimization

We used a hyperparameter optimization strategy to train models with a variety of architectures and training parameters. This was accomplished using the open-source Future Gadget Laboratory (https://github.com/Kaixhin/FGLab) framework. Table 1 describes the hyperparameters and their value ranges that were explored using a random search.

**Table 1:** Hyperparameters explored during model development and training.

Parameter	Values	Description
Arch	VGG16, VGG19, ResNet-50, Xception, Inception	Pretrained architecture on ImageNet
Pooling	Global average, global max, flatten	Pooling method after final filter layers
fc1	4, 8, 16, 32, 64, 128	Neuron count for first fully connected layer after pooling
fc2	0, 4, 8, 16, 32, 64, 128	Neuron count for the second fully connected layer
LR	0.001, 0.005, 0.01, 0.02	Learning rate
LR schedule	Constant, cyclic, plateau	Experiments with dynamic learning rates
Batch size	4, 8, 16, 32, 64, 128	Batch size for the training
Dropout	0, 0.25, 0.5, 0.75	Dropout setting applied to fully connected layers
Augmentation zoom	0, 0.25, 0.5, 0.75, 1.0	Maximum fractional zoom range for images. 1.0 = 100% increase in size
Augmentation shear	0, 0.1, 0.3, 0.5	Fractional affine shear for image augmentation generator
Augmentation rotation	0, 30, 45, 60, 90	Maximum rotational angle in degrees for image augmentation
Optimizer	sgd, adam, nadam, adadelta, rmsprop	Optimization algorithm used for training
Batch normalization	Yes/no	A batch normalization layer was optionally inserted before the pooling layer
ImgShape	256, 512, 1,024	The size of the downsampled image in pixels

### Model evaluation on internal test dataset

During model training, we evaluated model performance on the validation set every 10 training epochs. We computed the area under the receiver operating characteristic curve (AUC), sensitivity, specificity, and positive predictive value (PPV) of the entire validation set, and also log classification accuracies for each annotation class using scikit-learn (version 0.19.1). Receiver operating characteristic (ROC) curves were plotted using matplotlib (version 2.2.2). PPVs for lower prevalence scenarios were calculated based on each model’s observed sensitivity and specificity [19]. Our primary outcome was performance in distinguishing moderate or large pneumothorax from negative studies (containing no pneumothorax or trace pneumothorax), as this most closely matched our prespecified design requirements for prioritization of those acute pneumothorax cases most likely to be emergent based on size. We further tested our models using the full test set of images containing small, moderate, and large pneumothoraces as positive cases to more accurately approximate real-world clinical use.

### Model evaluation on external test dataset

We performed external evaluation of the models by evaluating performance on the National Institutes of Health (NIH) ChestX-ray14 set (available at https://nihcc.app.box.com/v/ChestXray-NIHCC), a publically available dataset of over 112,000 frontal chest radiographs accompanied by labels extracted from accompanying radiology reports using natural language processing. Since the images containing pneumothorax are not further classified by pneumothorax size, any label of “pneumothorax” in this set was considered a positive case. Confidence intervals for all reported measures were computed using the epiR (version 0.9–96) package in the R statistical computing environment (version 3.5.0).

## Results

### Model parameter optimization

While manipulation of some hyperparameters produced significant changes in model performance, other parameters produced reasonable models over a variety of settings. The 16 top-performing models (Table 2), for example, tended to favor a higher maximum zoom setting, low batch sizes, and low dropout fractions. However, the top models contained wide variation in the neuron counts of their fully-connected layers (fc1 and fc2), which did not appear to profoundly affect validation performance as assessed by AUC.

**Table 2:** Top 16 models classifying large and moderate pneumothorax, excluding small pneumothoraces in training.

Training				Validation				Arch	Layer neuron count		Batch size	Dropout	Pool	Augmentation		LR
AUC	Sens	Spec	PPV	AUC	Sens	Spec	PPV		fc1	fc2				Zoom	Shear	
0.95	0.85	0.90	0.44	0.94	0.79	0.91	0.43	VGG19	16	4	16	0	Flat	1	0.3	0.001
0.96	0.84	0.94	0.55	0.94	0.70	0.93	0.45	VGG16	32	8	16	0.25	Max	0.50	0.3	0.001
0.97	0.87	0.93	0.54	0.94	0.74	0.93	0.47	VGG19	16	16	16	0	Avg	0.50	0.3	0.01
0.98	0.88	0.97	0.71	0.94	0.69	0.97	0.64	Inception	64	32	16	0.25	Avg	0.50	0.5	0.02
0.97	0.87	0.95	0.61	0.93	0.70	0.94	0.51	Inception	4	4	16	0.50	Avg	1	0.5	0.001
0.93	0.75	0.93	0.51	0.93	0.68	0.94	0.49	VGG19	64	4	16	0.25	Avg	1	0.1	0.005
0.97	0.83	0.96	0.65	0.92	0.69	0.96	0.60	Inception	4	0	16	0.50	Max	0.75	0.3	0.02
0.97	0.81	0.96	0.64	0.92	0.63	0.96	0.60	Xception	4	0	4	0	Max	0.50	0.1	0.01
0.95	0.78	0.96	0.62	0.92	0.64	0.96	0.56	VGG19	32	8	4	0	Flat	0.50	0.1	0.001
0.95	0.68	0.98	0.75	0.92	0.55	0.97	0.64	ResNet	0	0	8	0.25	Max	0.75	0.1	0.001
0.94	0.79	0.94	0.54	0.92	0.72	0.94	0.52	Xception	16	8	4	0.25	Avg	0.75	0.1	0.02
0.95	0.77	0.96	0.64	0.92	0.64	0.96	0.60	VGG19	32	0	8	0.75	Flat	0.50	0.3	0.001
0.97	0.86	0.93	0.55	0.91	0.69	0.94	0.47	Xception	32	0	8	0	Flat	1	0.1	0.01
0.95	0.81	0.92	0.49	0.91	0.68	0.93	0.44	VGG16	64	8	16	0.25	Flat	1	0.1	0.001
0.94	0.67	0.96	0.61	0.91	0.60	0.96	0.58	ResNet	16	16	16	0.25	Avg	0.75	0.5	0.01
0.95	0.84	0.92	0.50	0.88	0.63	0.92	0.39	VGG19	32	16	16	0	Flat	0.75	0.1	0.005

Arch, architecture; AUC, area under the receiver operating characteristic curve; LR, learning rate; PPV, positive predictive value; Sens, sensitivity; Spec, specificity.

Both of our best models (described below) used batch normalization applied before pooling layers. Stochastic gradient descent was utilized as the optimizer, coupled with an initial learning rate of 0.02, stepped down in plateau fashion by a factor of 10 every 15 epochs. Training was stopped if no improvement was seen in the validation loss within 15 epochs following a decrease in learning rate.

### Model performance

We trained 7,475 models (representative model and training script code in S3 and S4 Texts) using random grid search for hyperparameter optimization. Attempts to initialize these models from random weights using the training set produced poor performance as evaluated by training loss. However, transfer learning using models pretrained on the ImageNet dataset was much more successful. We identified 4 top-performing models based on the AUC obtained for the validation set images. These 4 models all had a validation AUC of 0.94. Prior to evaluation on the test set (which had been kept completely separate from the training process), we selected 2 models of the 4 as our “best” models. The first (VGG19-based, hereafter the “high sensitivity model”) showed the best sensitivity (0.79) on the validation data, and the second (Inception-based, hereafter the “high specificity model”) showed the best specificity (0.97).

**Fig 1:**
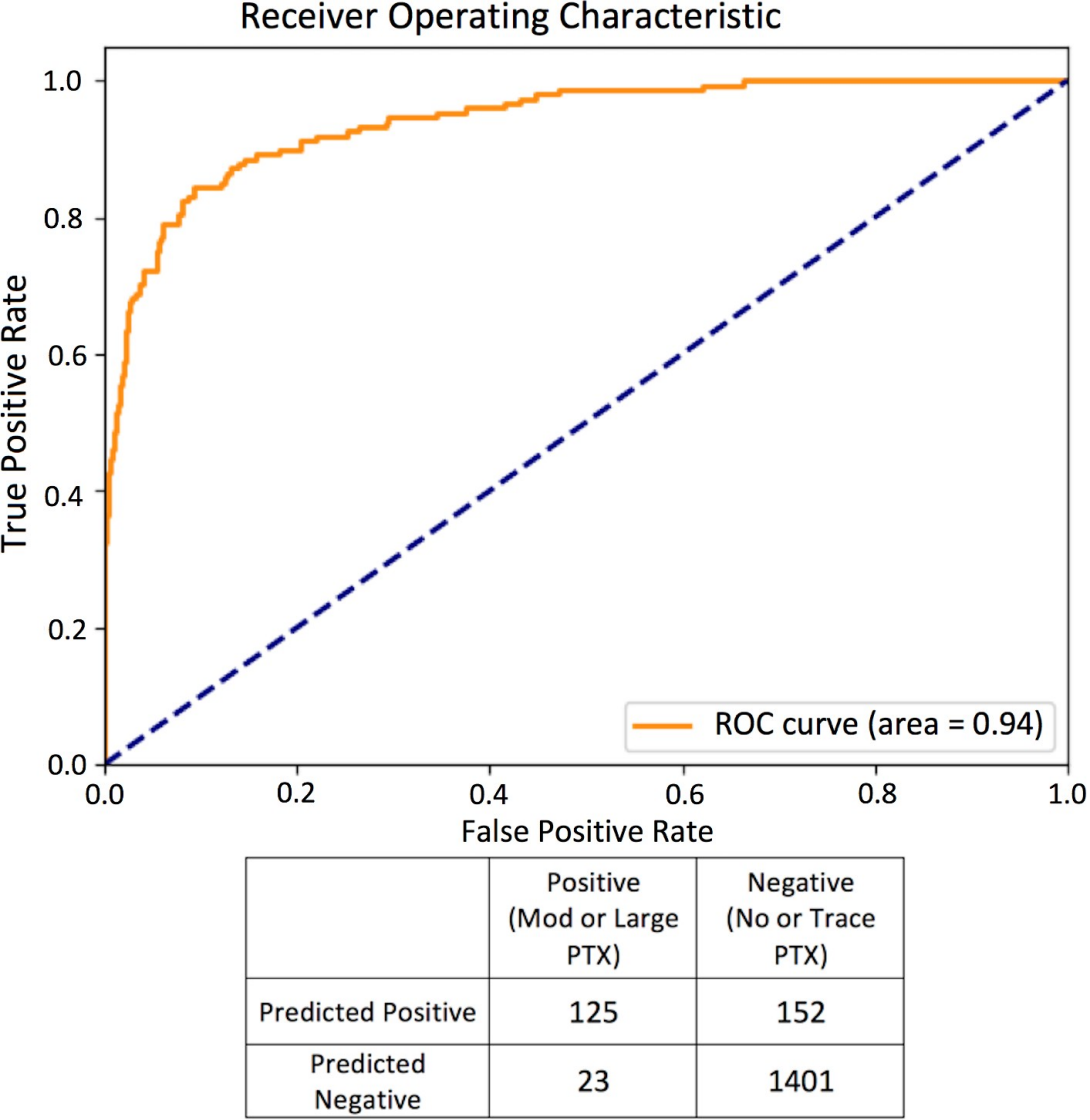
Diagnostic performance ROC curve and confusion matrix of most sensitive top model evaluated on test set (small pneumothoraces excluded). Mod, moderate; PTX, pneumothorax; ROC, receiver operating characteristic.

**Fig 2:**
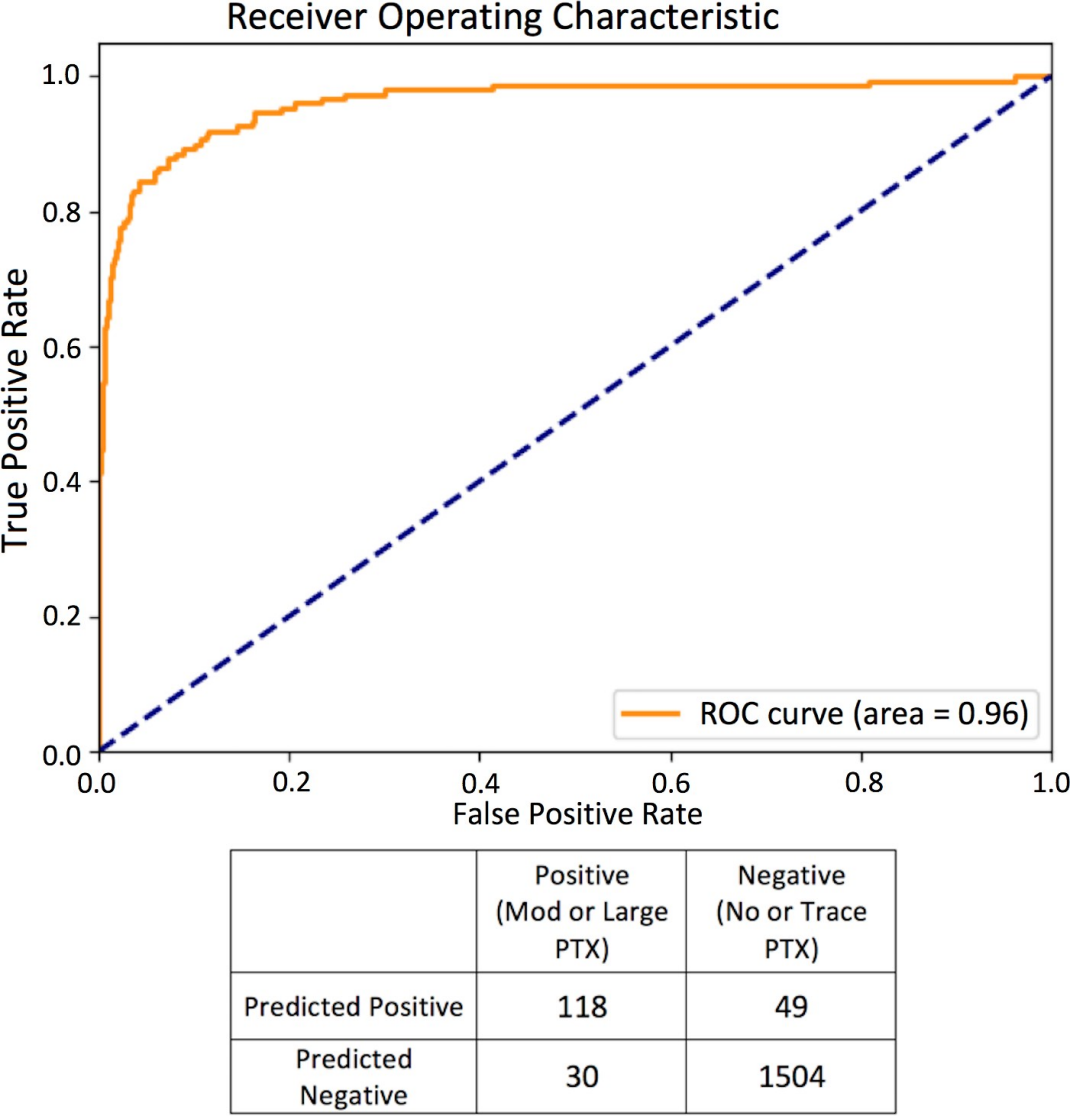
Diagnostic performance ROC curve and confusion matrix of most specific top model evaluated on test set (small pneumothoraces excluded). Mod, moderate; PTX, pneumothorax; ROC, receiver operating characteristic.

Evaluated on the test data, the high sensitivity model showed a sensitivity of 0.84 (95% CI 0.78–0.90), specificity of 0.90 (95% CI 0.89–0.92), and AUC of 0.94 (Fig 1). The high specificity model showed a sensitivity of 0.80 (95% CI 0.72–0.86), specificity of 0.97 (95% CI 0.96–0.98), and AUC of 0.96 (Fig 2). The PPVs for these 2 models on the test set were 0.45 (95% CI 0.39–0.51) and 0.71 (95% CI 0.63–0.77), respectively. Since pneumothorax is a rare event overall but has a very heterogeneous incidence based on the patient population being evaluated (e.g., it is rare in the population at large but as high as 15% in mechanically ventilated patients [20]), PPVs were calculated for the high sensitivity and high specificity models using an expected prevalence of 1%, yielding values of 7.8% and 21.2%, respectively.

When evaluated on the full internal test set of images containing small, moderate, and large pneumothoraces as positive cases, the overall sensitivity of our models declined to 0.55 (high sensitivity model; Table 3) and 0.45 (high specificity model; Table 4), which was expected since the models had not been trained using small pneumothorax images. However, sensitivity for large pneumothorax remained 1.00 for the high sensitivity model and 0.88 for the high specificity model, and sensitivity for moderate pneumothorax remained 0.82 for the high sensitivity model and 0.78 for the high specificity model. Specificity overall remained quite high, at 0.90 (high sensitivity model) and 0.97 (high specificity model). When applied to a theoretical pneumothorax prevalence of 1%, PPVs were calculated at 5.3% (high sensitivity model) and 12.6% (high specificity model). Using the full test dataset, the high sensitivity model AUC dropped from 0.94 to 0.82, and the high specificity model AUC dropped from 0.96 to 0.86. These metrics were calculated to facilitate comparison of performance on our internal test set against performance on the external NIH dataset, which contains pneumothoraces of all sizes, not annotated based on size.

**Table 3:** High sensitivity model test set performance, stratified by pneumothorax size.

Annotation	Predicted correctly	Predicted incorrectly	Percentage correct	95% CI
Negative	1,239	113	92	90–93
Trace PTX	162	39	81	75–85
Small PTX	113	176	39	34–45
Moderate PTX	101	23	81	73–87
Large PTX	24	0	100	86–100

PTX, pneumothorax.

**Table 4:** High specificity model test set performance, stratified by pneumothorax size.

Annotation	Predicted correctly	Predicted incorrectly	Percentage correct	95% CI
Negative	1,311	41	97	96–98
Trace PTX	193	8	96	92–98
Small PTX	80	209	28	23–33
Moderate PTX	97	27	78	70–85
Large PTX	21	3	88	69–96

PTX, pneumothorax.

When evaluated against the NIH ChestX-ray14 set (Table 5), our models did show some decline in performance compared to the test done with our internal dataset containing small, moderate, and large pneumothoraces. The high sensitivity model showed a performance of 0.49 sensitivity and 0.85 specificity on the NIH set as opposed to 0.55 sensitivity and 0.90 specificity for our internal test set. The high specificity model showed decline in sensitivity to 0.28 on the NIH set as opposed to 0.45 on our internal set, but specificity remained essentially unchanged at 0.97 on the NIH set compared to 0.97 on the internal set.

**Table 5:** Performance of the models on the NIH ChestX-ray14 external dataset.

Measure	High sensitivity model		High specificity model	
	Positive (any PTX)	Negative (no PTX)	Positive (any PTX)	Negative (no PTX)
Predicted positive	2,602	16,269	1,481	3,737
Predicted negative	2,700	90,549	3,821	103,081
Sensitivity	0.49		0.28	
Specificity	0.85		0.97	
PPV	0.14		0.28	
NPV	0.97		0.96	
AUC	0.75		0.75	

AUC, area under the receiver operating characteristic curve; NIH, National Institutes of Health; NPV, negative predictive value; PPV, positive predictive value; PTX, pneumothorax.

## Discussion

We created automated models that had high AUC and were sensitive to large and moderate pneumothoraces while retaining high specificity when evaluated on our internal test set. In particular, the high specificity model (specificity 0.97) produced a PPV of 12.5% for the scenario in which pneumothorax has a prevalence of 1% (including small, moderate, and large pneumothoraces). This performance profile matches what is required for prioritization of low-prevalence findings. While high sensitivity is of course desirable, for our selected use case of triaging larger, potentially more acutely clinically significant pneumothoraces at times when review may be delayed (i.e., overnight), we felt it important that PPV remain high enough that there will not be too many false positives, since this would increase alert fatigue, and clinical radiologists might ignore the findings of the algorithm. With a PPV of 12.5%, a radiologist need only review approximately 8 radiographs for every positive case. However, it is important to make clear that this algorithm is not intended to be relied upon to detect small pneumothoraces (based on our experimental design and training method), and that some moderate and large pneumothoraces may still be missed. In keeping with our research aim, this is meant to be a prioritization and triaging tool for potential emergencies rather than a substitute for careful image review and diagnosis rendered by a human radiologist.

For human radiologists, detection of pneumothorax has been shown to be affected by both image resolution and luminance [21]. Currently, significant downsampling of the image is required for algorithm training on a full X-ray image due to memory and free parameter constraints. High-resolution images also require more computational power and memory than may be available in devices (such as portable X-ray devices) that might benefit from the deployment of early screening algorithms to the platform. While we hypothesize that the classifiers’ limited ability to detect small pneumothoraces may be partly due to resolution limitations, it is not fully explained by this. Nevertheless, using downsampled images, the classifiers perform well in identifying large and moderate pneumothoraces, which we suggest are the most clinically emergent findings due to the mechanism by which pneumothorax causes respiratory and circulatory collapse.

Automated and semi-automated models have been developed for the detection of pneumothorax previously, using both traditional image-analysis techniques and machine learning and deep learning approaches. For example, a method using traditional techniques to identify pleural lines following identification of posterior rib lines yielded a sensitivity for pneumothorax of 77% on a small set of images (22 positive and 28 negative images). However, this method produced a per-image false-positive rate of 0.44 [22]. More recently, machine learning and deep learning approaches have been used to address this problem. Several groups have focused on developing classifiers using the NIH ChestX-ray8 dataset, an earlier subset of the ChestX-ray14 set, which contains 108,948 frontal chest X-rays labeled with 8 different disease classifiers on the basis of mining text reports. Using the text-based labeling method, 2,793 images were deemed positive for pneumothorax, and a classifier developed using the ResNet architecture achieved an AUC of 0.789 [23]. A deeper network architecture trained on the subsequent ChestX-ray14 dataset produced an AUC for pneumothorax of 0.889 [24]. Cicero et al. utilized a pretrained Inception architecture and retrained on a set of approximately 35,000 radiographs (downsampled to 256 × 256 pixels) from their institution classified into 5 categories including pneumothorax (1,299 images), labeled by mining the clinical report text [25]. Interestingly, they excluded images where the report labeled the pneumothorax as “tiny, trace, small, decreased or improved.” This likely produced a dataset reasonably similar to our training set, which also excludes small pneumothorax. Of note, Cicero et al. did not explicitly address chest tubes as a confounding finding in their data, so it is unclear whether their dataset and ours have similar makeup and distribution with regard to this feature, and unknown what effect this may have had on model performance.

Using the ChestX-ray14 dataset for external testing of our models, we observed some decline in performance of our high sensitivity model compared to testing performed on our own held-out test set (with small pneumothoraces included), with an AUC of 0.82 (internal) versus an AUC of 0.75 (NIH). Our high specificity model exhibited a decrease in AUC from 0.86 (internal) to 0.75 (NIH); however, specificity remained high and unchanged (0.97 internal versus 0.97 NIH). Some decline in performance seen on this task may also be related to the fact that the NIH set does not attempt to stratify pneumothorax on the basis of size, and so may contain larger numbers of small and trace pneumothoraces. Further, while it is a very valuable resource to have such a large dataset publicly available, questions have been raised as to the accuracy of the annotations obtained through text mining reports in this set; in the case of pneumothorax, agreement between the NIH label and radiologist review has been reported to be only approximately 60% [26]. Errors in these annotations may artificially decrease the apparent performance of algorithms on this dataset.

Our work expands and improves upon prior work by producing a very high level of performance (AUC of 0.94 for detection of moderate and large pneumothoraces, 0.86 for detection of pneumothoraces overall when our internal test set included small pneumothoraces) while generating acceptably low levels of false positives when applied to a low-prevalence finding such as pneumothorax. Further, our work is based on a new dataset of over 13,000 images (of which 3,107 are positive for pneumothorax) created by direct visual reannotation of each image by trained radiologists, further refined through consensus labeling for the positive cases. While further performance improvements are likely to be gained through model refinement and the use of new model architectures, our experiments in the development of these models agree with others’ assertions [27] that dataset size and quality remain a key factor in the production of clinically useful classifiers that generalize well to chest X-rays obtained in a variety of clinical scenarios and on a range of patients, from healthy outpatients to the sickest patients in an ICU setting.

Our work has several limitations. First, it is important to restate that our intent is to create a prioritization tool for potentially emergent pneumothorax. In the current state that exists at our institution and many others, no automated analysis of these images means that no images are flagged for priority review. With the use of this algorithm, we expect that the majority of moderate and large pneumothoraces would be prioritized for expedited review, but since the model sensitivity is not 100%, some moderate and large pneumothoraces would likely go undetected by the algorithm, in addition to the majority of small pneumothoraces, which were not represented in the model training set. Second, although we intentionally included frontal chest X-rays from as many different clinical settings as possible (e.g., outpatient clinic, emergency department, and ICU), the training and test data, and the highest performing scores described above, are based on data from a single institution. Further, “ground truth” for this study relies on the consensus opinion of the radiologists performing annotation of the images; no clinical follow-up or additional testing was performed to confirm diagnoses. Finally, although our results suggest that our classifiers may be valuable tools for triage and prioritization of studies with a critical finding such as pneumothorax, they have not yet been prospectively tested in a clinical environment, and since the composition and structure of the external NIH dataset used for external testing does not exactly match that of our internal data, it is difficult to predict exactly how well the algorithms would transfer to other institutions.

In summary, we have developed automated image classifiers that detect clinically significant pneumothorax (moderate and large in size as determined by radiologist consensus read) at high levels of performance within a single site, while maintaining a reasonable false-positive rate. These classifiers may have practical value as triage tools for identifying and prioritizing studies for expedited review, while minimizing the number of negative studies that must be reviewed as urgent in order to identify the true positives. These classifiers were developed using a training dataset of over 13,000 images where ground truth was established by direct visual reannotation of each study by trained radiologists. We hope that this work represents first steps into the development and deployment of truly useful artificial intelligence tools in the medical imaging space, and that implementation of such algorithms can improve the speed and quality of care delivered across a variety of healthcare settings.

## Supporting information

S1 Fig Representative details from the image set provided to annotators showing examples of the 4 qualitative classifications of pneumothorax size. (TIFF)

S1 Text Description of pneumothorax classification rubric used by annotators. (DOCX)

S2 Text Prespecified research design (September 2016). (DOCX)

S3 Text Representative Python code for model creation. (TXT)

S4 Text Representative Python code for model training. (TXT)

## Abbreviations

AUC: area under the receiver operating characteristic curve
ICU: intensive care unit
NIH: National Institutes of Health
PPV: positive predictive value
ROC: receiver operating characteristic
