# Supplementary figures and images for: Automated detection of moderate and large pneumothorax on frontal chest X-rays using deep convolutional neural networks: A retrospective study
Source: PLoS Med. 2018 Nov 20;15(11):e1002697. doi: 10.1371/journal.pmed.1002697 (PMC6245672; doi:10.1371/journal.pmed.1002697)

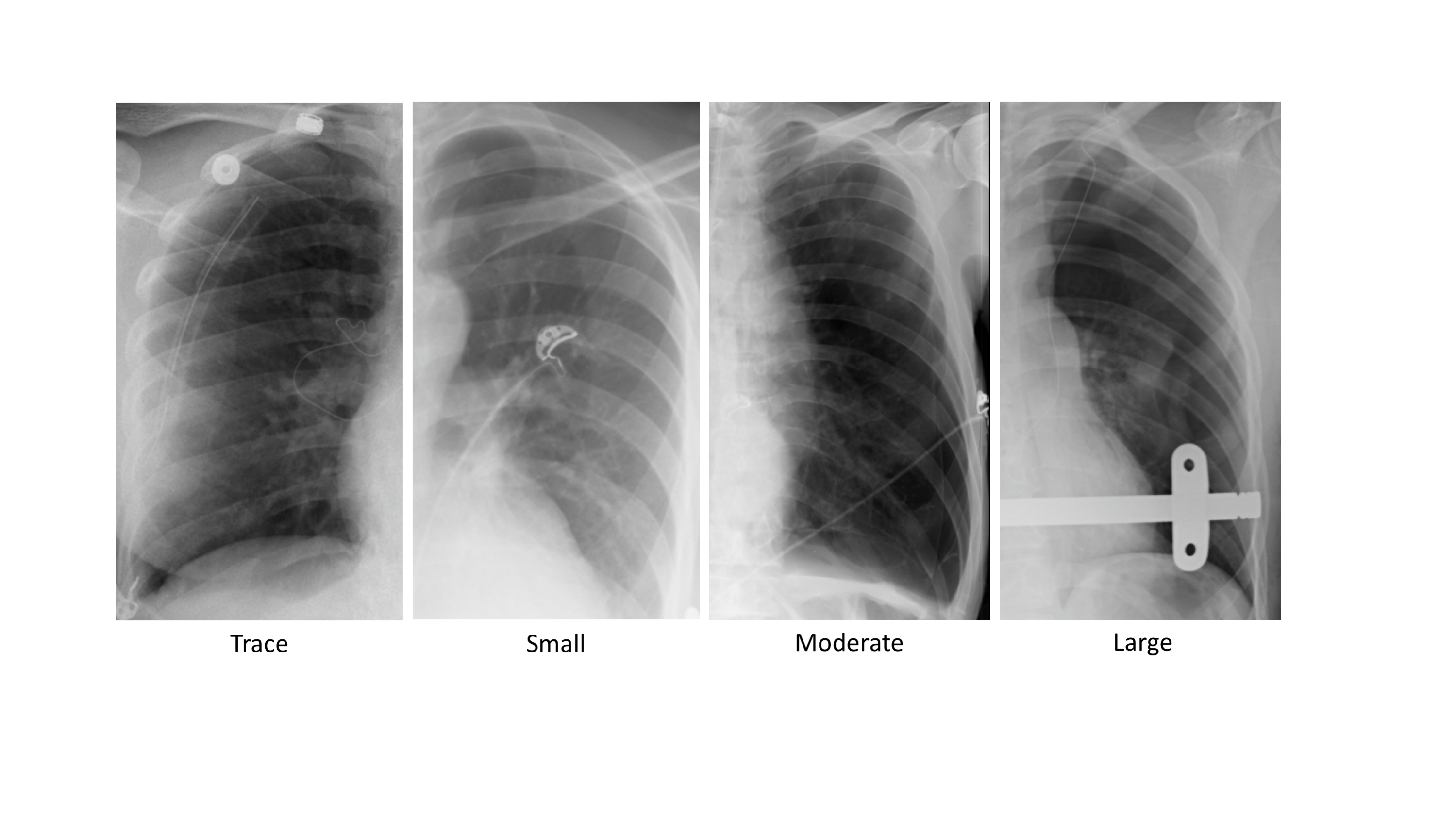

Supplement: S1 Fig — (TIFF) [file pmed.1002697.s001.tiff]
