# Supplementary material for: Automated detection of moderate and large pneumothorax on frontal chest X-rays using deep convolutional neural networks: A retrospective study
Source: PLoS Med. 2018 Nov 20;15(11):e1002697. doi: 10.1371/journal.pmed.1002697 (PMC6245672; doi:10.1371/journal.pmed.1002697)
Supplement: S1 Text — (DOCX) [file pmed.1002697.s002.docx]

**S1 Text. Description of Pneumothorax Classification Rubric Used by Annotators**

To achieve a reasonable degree of uniformity among annotators when classifying images with pneumothorax, annotators were given several example images of the four qualitative classifications of pneumothorax assigned to this data set (see example images in Fig S1 below). Images assigned “Trace” showed a discrete visceral pleural line that was separate from but very closely apposed to the parietal pleura. Those assigned “Small” demonstrated up to approximately 1 cm separation between visceral and parietal pleura and was typically confined to one lobe of region of the lung (e.g. apex, lateral, medial, base). Images classified as “Moderate” typically showed larger separation between visceral and parietal pleura (approximately 1-2 cm), and often extended to involve more than one lobe or area of the lung. Pneumothoraces classified as “Large” generally showed wide (greater than 2 cm) separation between the parietal and visceral pleura, and involved multiple areas of the affected lung.
