# Supplementary material for: Automated detection of moderate and large pneumothorax on frontal chest X-rays using deep convolutional neural networks: A retrospective study
Source: PLoS Med. 2018 Nov 20;15(11):e1002697. doi: 10.1371/journal.pmed.1002697 (PMC6245672; doi:10.1371/journal.pmed.1002697)
Supplement: S2 Text — (DOCX) [file pmed.1002697.s003.docx]

UCSF: SOW #1 Design Session

**App:** “Pneumothorax Detection”

**Description from SOW:** Pneumothorax creates life-threatening complications that require urgent intervention. The majority of cases are attributed to trauma or iatrogenic causes, while patients with pre-existing conditions, including emphysema, cystic fibrosis, lung cancer, HIV may also develop pneumothorax.^[[1]](#footnote-1)^ Diagnosis is made with a plain chest radiograph followed by immediate chest tube placement for medium to large pneumothoraces. This highlights the importance of getting the x-ray performed and reported as quickly as possible. With some US data suggesting a median turnaround time of over 1 hour to perform and interpret an x-ray in the emergency room setting, this may result in an unacceptable delay in treating pneumothorax patients.^[[2]](#footnote-2)^ Algorithm-based triage for the reporting radiologist and point of care decision support for the provider/care team is a highly scalable and replicable way to save time and lives where radiologist capacity is limited or nonexistent.

| **Use Case #1: Automated Triage of Pneumothorax +ve CXRs** | |
| --- | --- |
| What is the key problem the use case is trying to solve? | Current system workflows do not support radiologists to quickly and correctly triage the read of chest x-rays (CXRs) on their worklists that contain evidence of “medium” to “large” pneumothoraces. If the study is not prioritized, the time from image generation, to radiologist interpretation and eventual treatment may be delayed, negatively impacting patient outcomes. |
| What is the proposed solution? | Application of a deep learning “Pneumothorax Detection” algorithm to designated CXRs immediately after image acquisition that aims to: 1) quickly identify the likely presence or absence of a “medium” to “large” pneumothorax; 2) alert the reporting radiologist to prioritize the read and report of CXRs in the worklist queue that the algorithm has confirmed as having radiographic evidence of a “medium” to “large” pneumothorax; 3) notify the referring provider/care team that a “medium” to “large” pneumothorax has been detected by the algorithm and will be prioritized by the duty radiologist. It is anticipated that the algorithm will exist in the cloud and/or on the imaging device, but this remains to be determined. |
| Who will benefit from the new solution? | - **Radiologists *(or “Other Radiology Staff” qualified to read and report CXRs)*:** The solution enables radiologists to reduce the time to read and report CXRs in the worklist with radiographic evidence of “medium” to “large” pneumothorax. Allows rads to bring greater value to the overall care pathway for this patient cohort. - **Referring provider/Care team:** The solution enables the referring provider to have a more timely result from the radiologist for patients whose CXRs show radiographic evidence of “medium” to “large” pneumothorax. - **Patient:** The solution enables patients with a “medium” to “large” pneumothorax on CXR to potentially receive a more prompt diagnosis. |

1. Noppen M, De Keukeleire T. Pneumothorax. *Respiration.* 2008;76(2):121-127. [↑](#footnote-ref-1)
2. Pines JM, Morton MJ, Datner EM, Hollander JE. Systematic delays in antibiotic administration in the emergency department for adult patients admitted with pneumonia. *Academic emergency medicine.* 2006;13(9):939-945. [↑](#footnote-ref-2)
